# Supplementary figures and images for: RNA localization mechanisms transcend cell morphology
Source: eLife. 2023 Mar 3;12:e80040. doi: 10.7554/eLife.80040 (PMC9984196; doi:10.7554/eLife.80040)

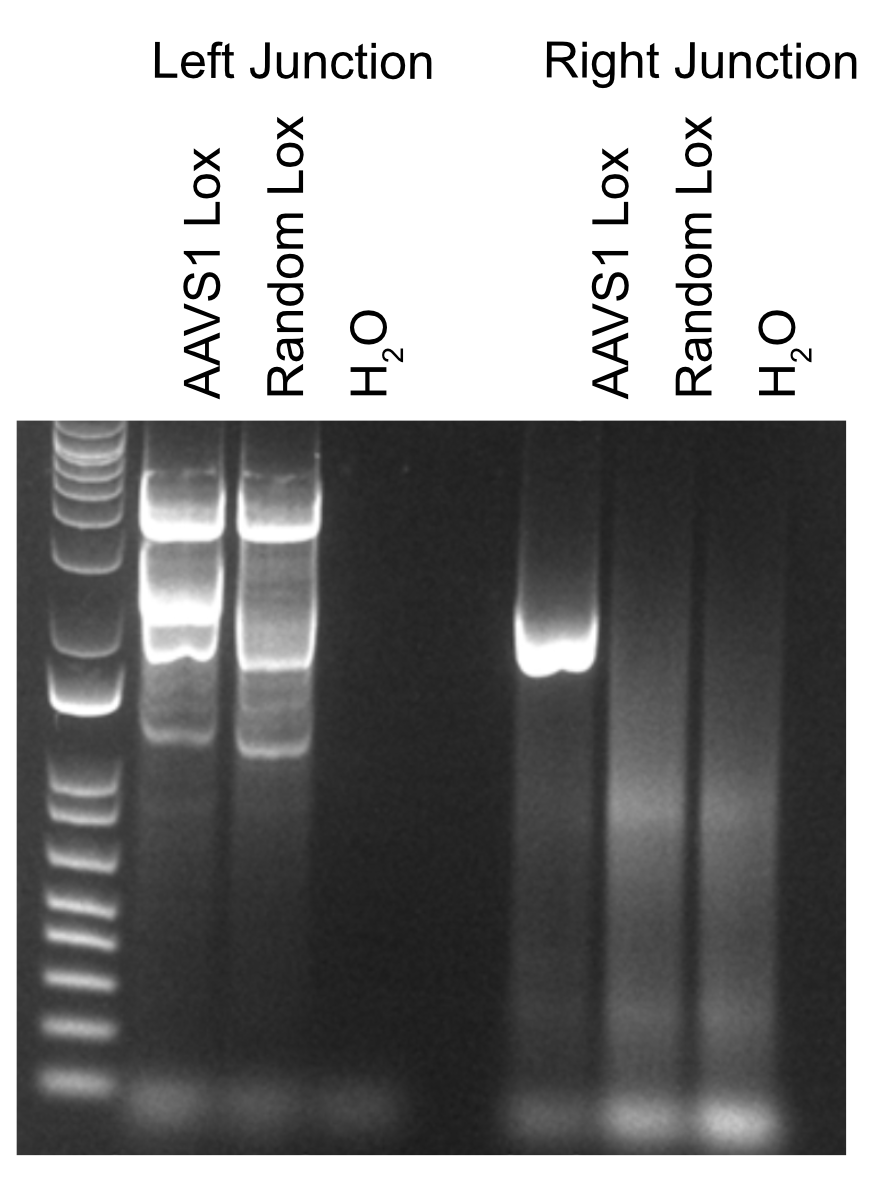

Supplement: Figure 1—figure supplement 2—source data 1. — Where targeted insertion is performed (AAVS1 lox) a unique band is observed. When the LoxP cassette is not targeted (Random Lox) we do not observe specific insertion into the AAVS1 site. A no template water control is also included. The ladder used is a 1 kb Plus ladder (NEB N3200). [file elife-80040-fig1-figsupp2-data1.zip › figure1-figuresupp2-sourcedata1/figure 1-figure supplement 2-source data 1-anno.png]

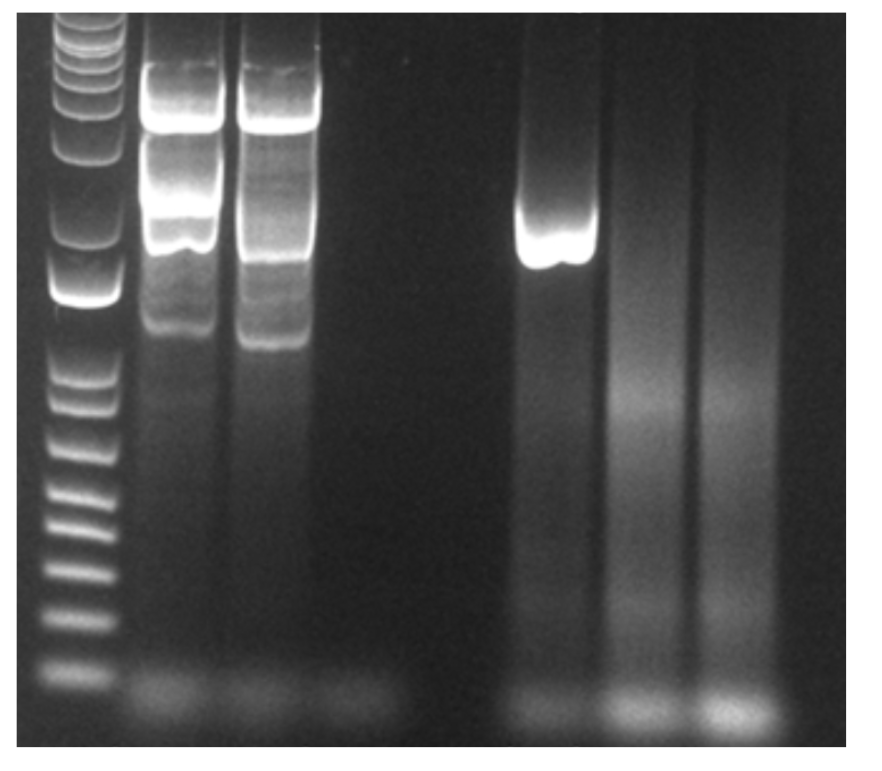

Supplement: Figure 1—figure supplement 2—source data 1. — Where targeted insertion is performed (AAVS1 lox) a unique band is observed. When the LoxP cassette is not targeted (Random Lox) we do not observe specific insertion into the AAVS1 site. A no template water control is also included. The ladder used is a 1 kb Plus ladder (NEB N3200). [file elife-80040-fig1-figsupp2-data1.zip › figure1-figuresupp2-sourcedata1/figure 1-figure supplement 2-source data 1.png]

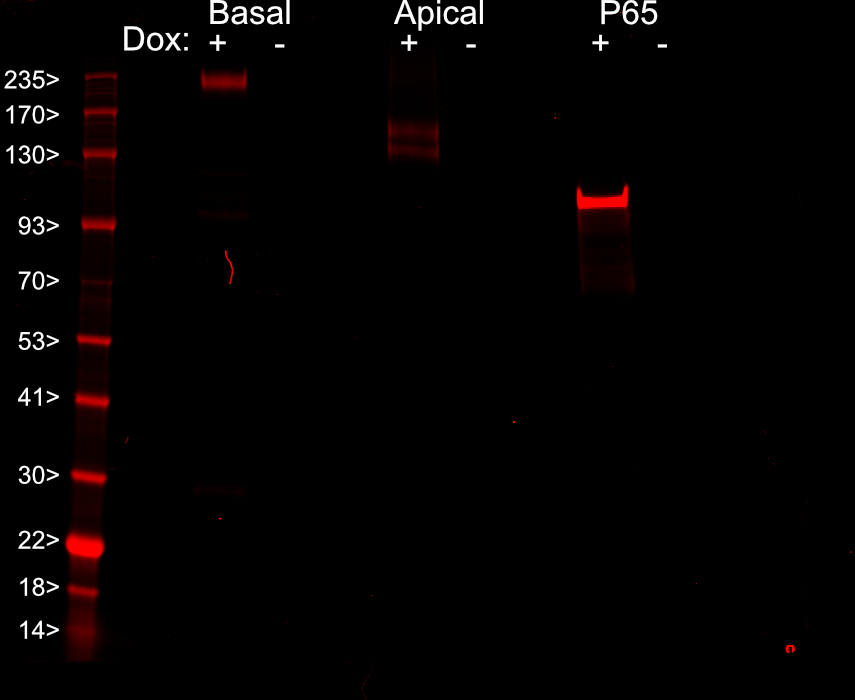

Supplement: Figure 1—figure supplement 4—source data 1. — The ladder is a broad-spectrum protein ladder (Fisher Scientific PI26623). [file elife-80040-fig1-figsupp4-data1.zip › figure1-figuresupp4-sourcedata1/figure 1-figure supplement 4-source data 1-anno.tiff]

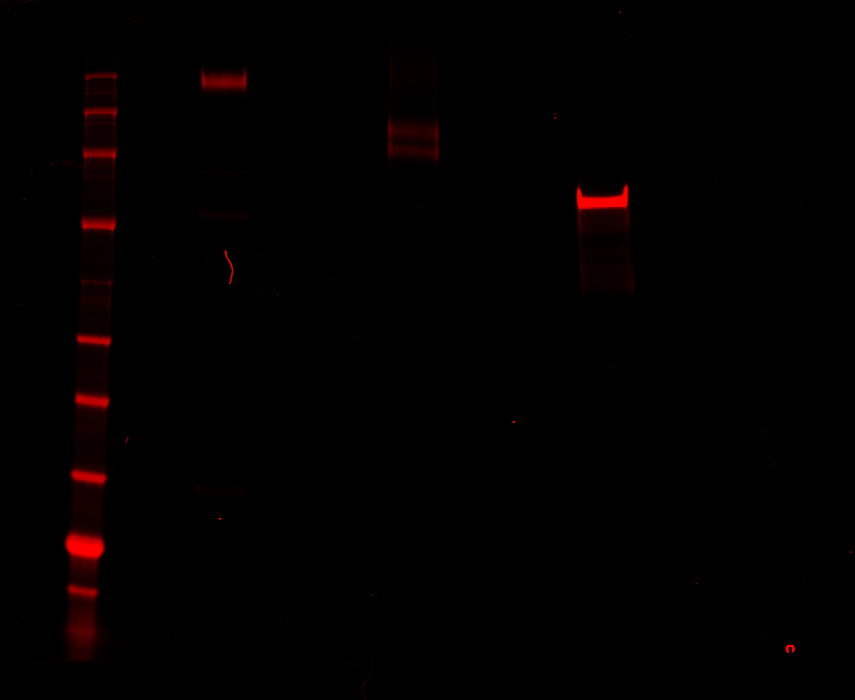

Supplement: Figure 1—figure supplement 4—source data 1. — The ladder is a broad-spectrum protein ladder (Fisher Scientific PI26623). [file elife-80040-fig1-figsupp4-data1.zip › figure1-figuresupp4-sourcedata1/figure 1-figure supplement 4-source data 1.tif]

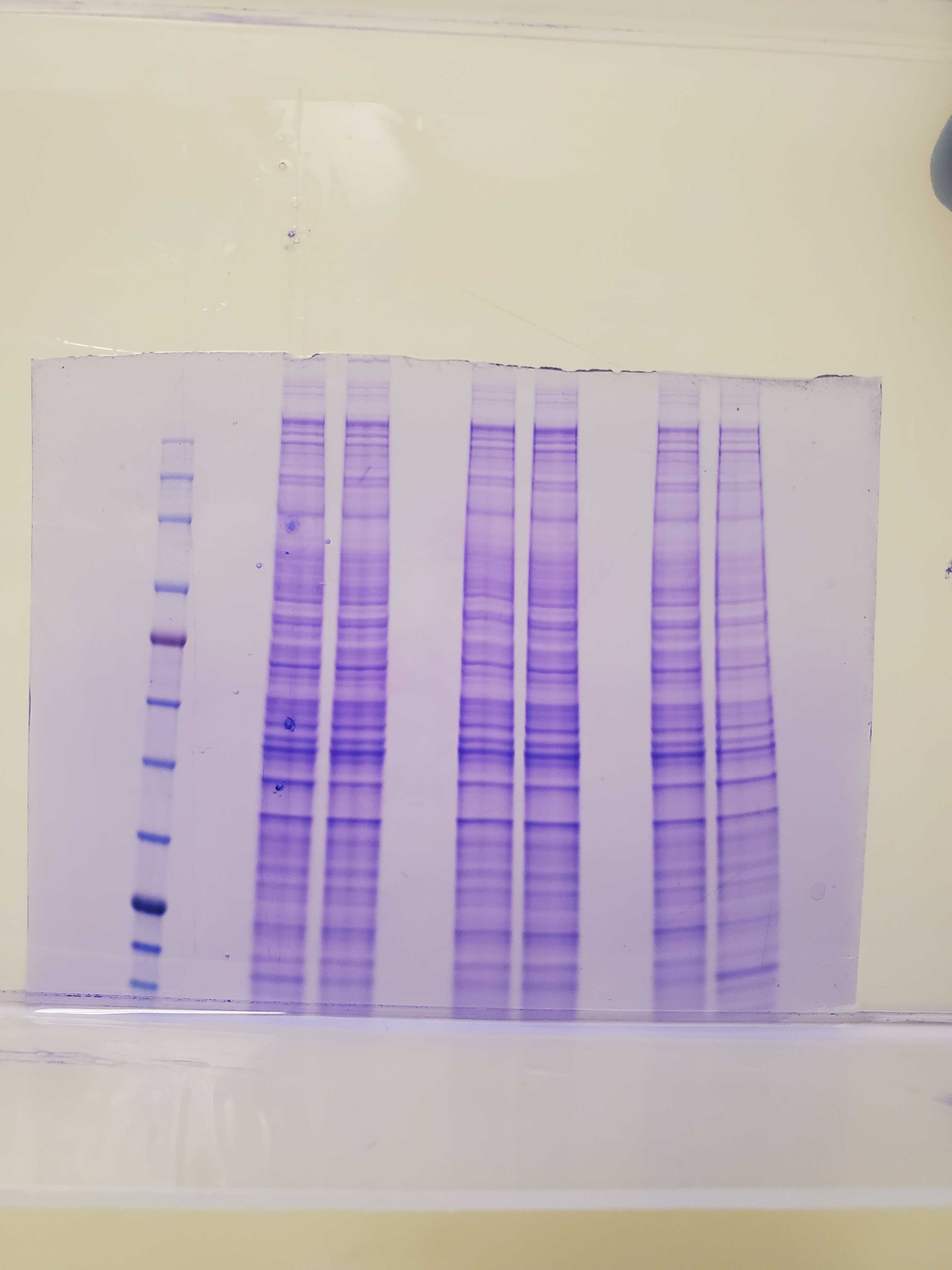

Supplement: Figure 1—figure supplement 4—source data 2. — The ladder is a broad-spectrum protein ladder (Fisher Scientific PI26623). [file elife-80040-fig1-figsupp4-data2.zip › figure1-figuresupp4-sourcedata2/figure 1-figure supplement 4-source data 2.tif]

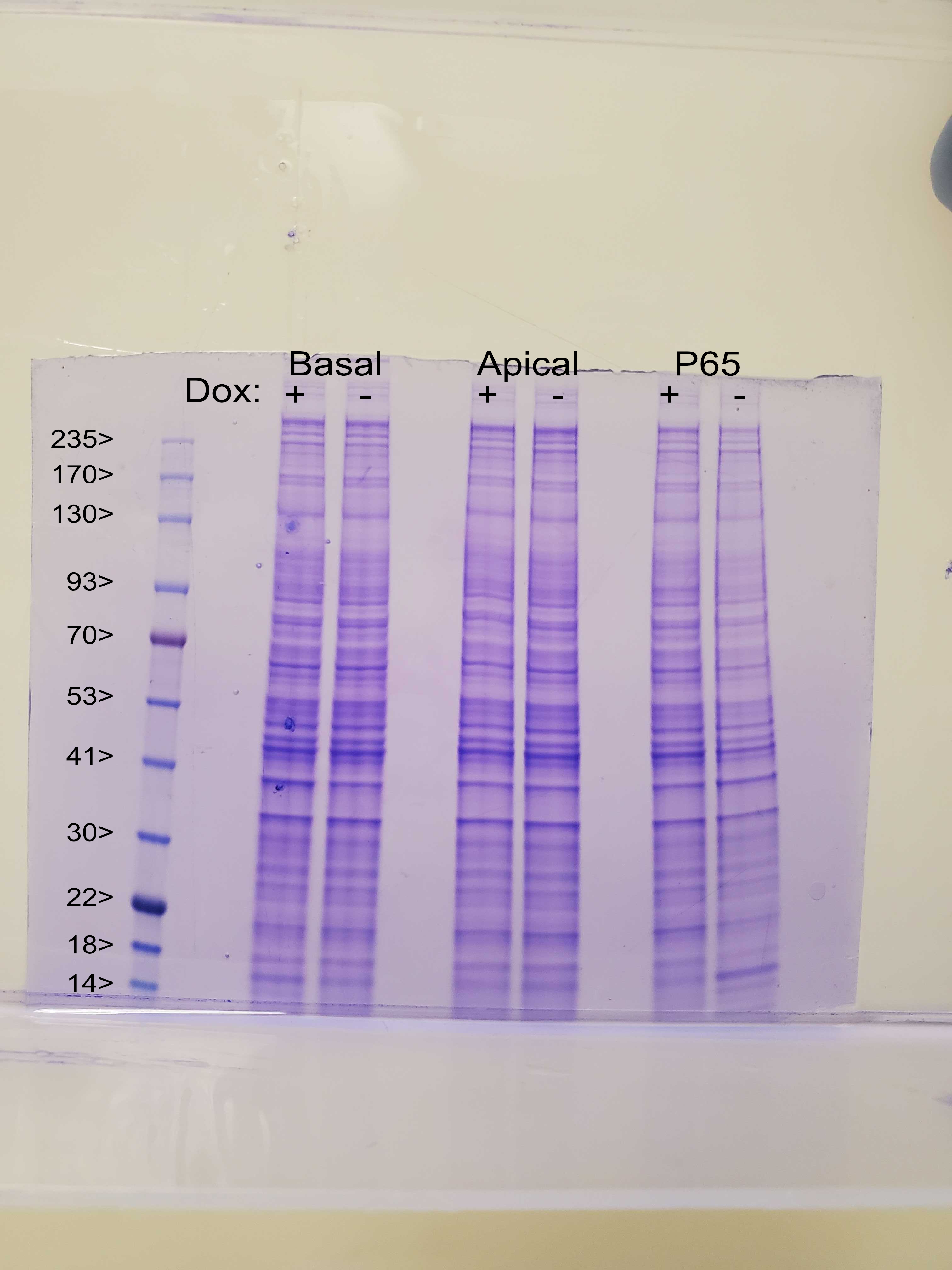

Supplement: Figure 1—figure supplement 4—source data 2. — The ladder is a broad-spectrum protein ladder (Fisher Scientific PI26623). [file elife-80040-fig1-figsupp4-data2.zip › figure1-figuresupp4-sourcedata2/figure 1-figure supplement 4-source data 2-anno.tiff]

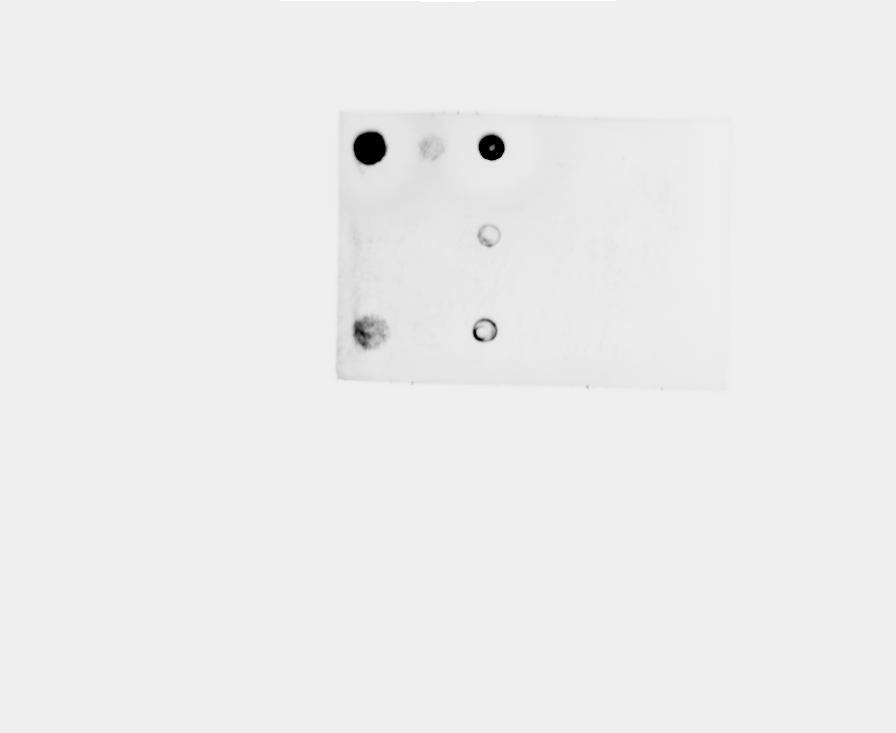

Supplement: Figure 1—figure supplement 6—source data 1. — Biotinylated RNA is visualized with Streptavidin HRP. [file elife-80040-fig1-figsupp6-data1.zip › figure1-figuresupp6-sourcedata1/figure 1-figure supplement 6-source data 1.tif]

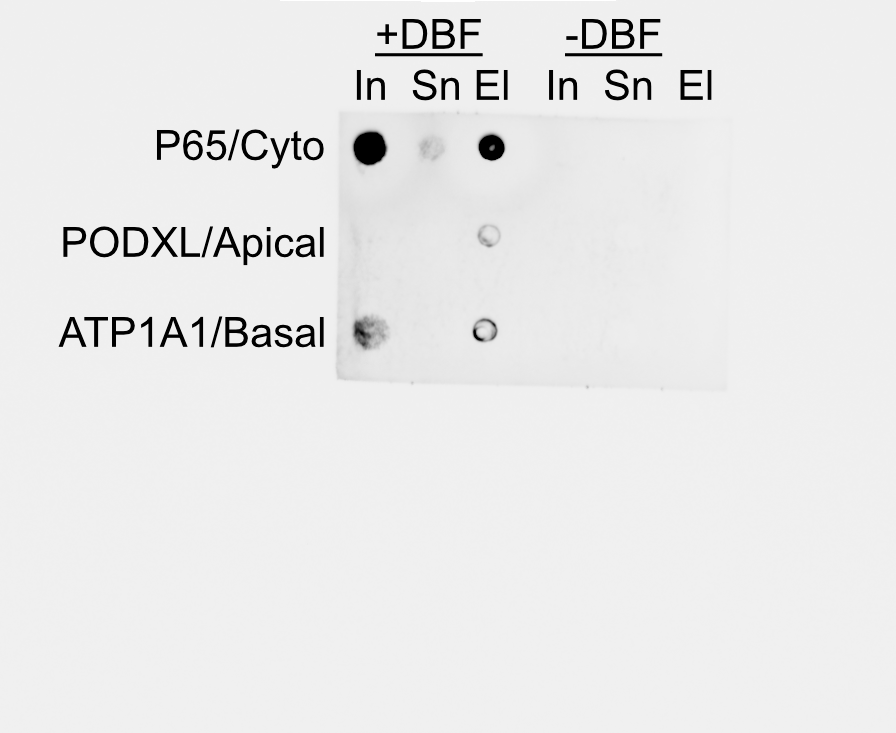

Supplement: Figure 1—figure supplement 6—source data 1. — Biotinylated RNA is visualized with Streptavidin HRP. [file elife-80040-fig1-figsupp6-data1.zip › figure1-figuresupp6-sourcedata1/figure 1-figure supplement 6-source data 1-anno.tiff]

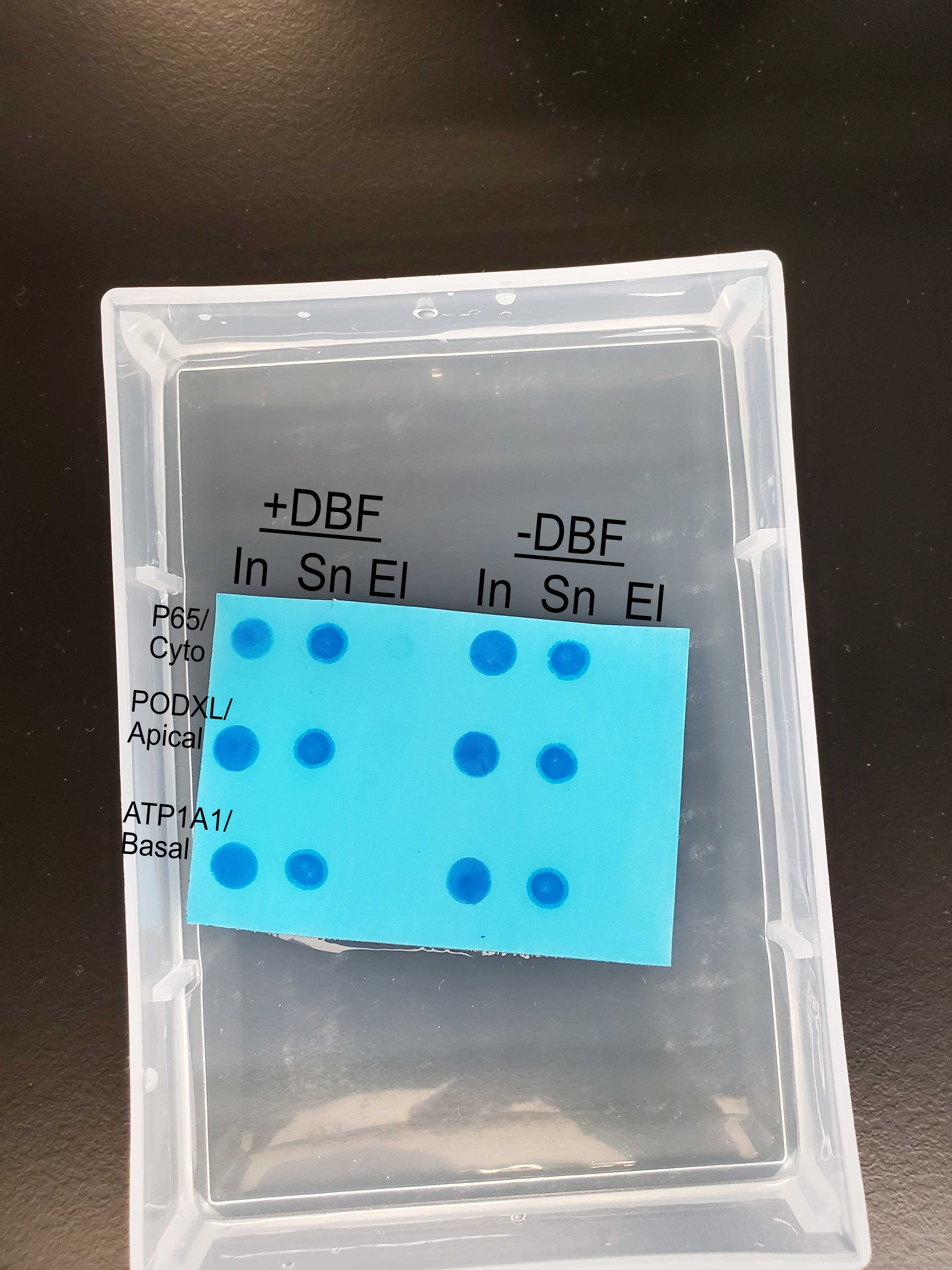

Supplement: Figure 1—figure supplement 6—source data 2. — Total RNA is visualized with 1% Methylene Blue. [file elife-80040-fig1-figsupp6-data2.zip › figure1-figuresupp6-sourcedata2/figure 1-figure supplement 6-source data 2-anno.tiff]

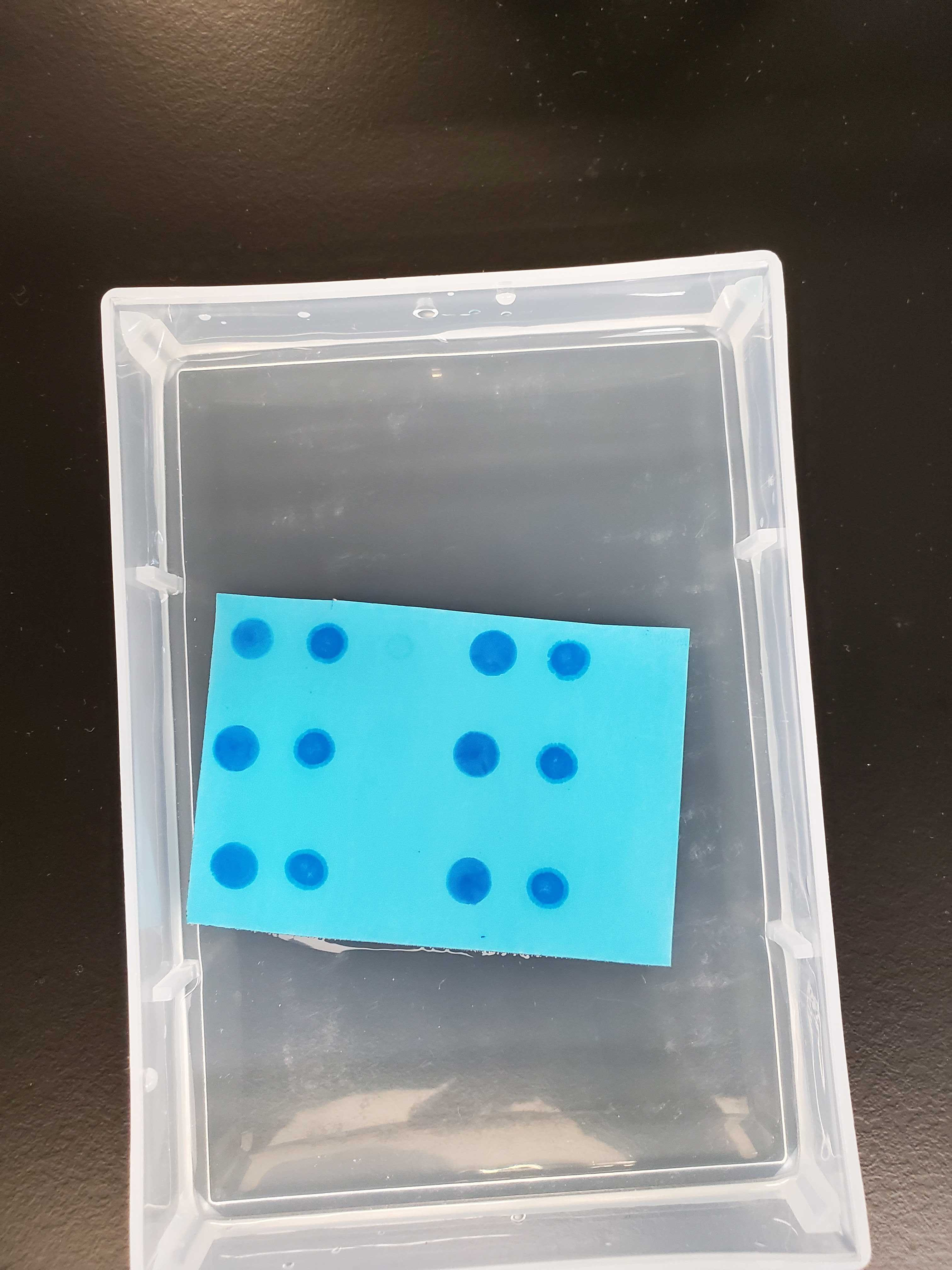

Supplement: Figure 1—figure supplement 6—source data 2. — Total RNA is visualized with 1% Methylene Blue. [file elife-80040-fig1-figsupp6-data2.zip › figure1-figuresupp6-sourcedata2/figure 1-figure supplement 6-source data 2.jpg]

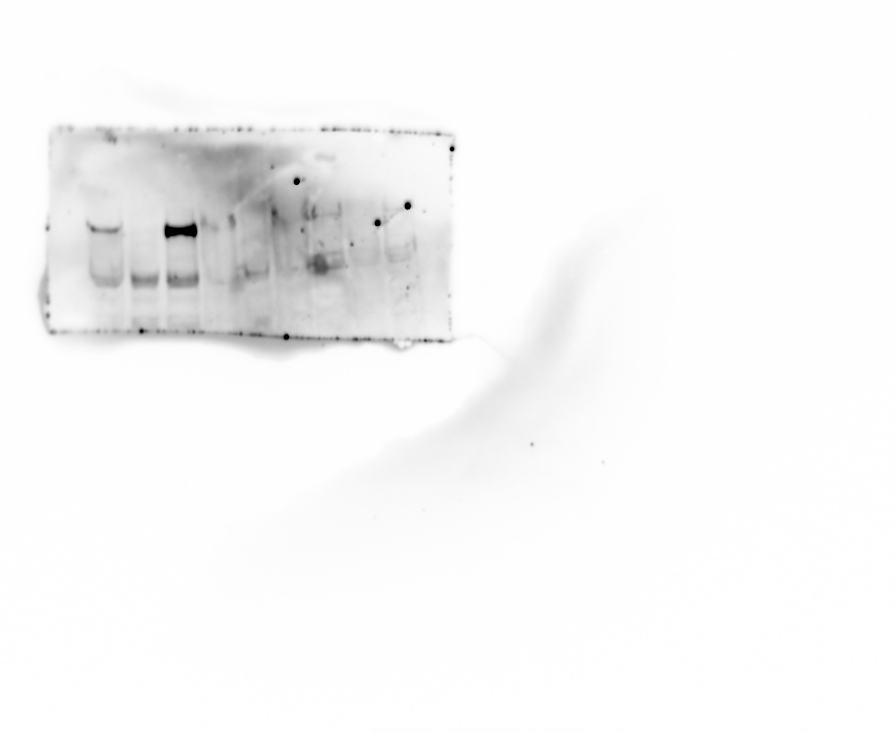

Supplement: Figure 4—source data 1. — LARP1 expression was visualized with a mouse monoclonal anti LARP1 antibody. [file elife-80040-fig4-data1.zip › figure4-sourcedata1/figure 4-source data 1.tif]

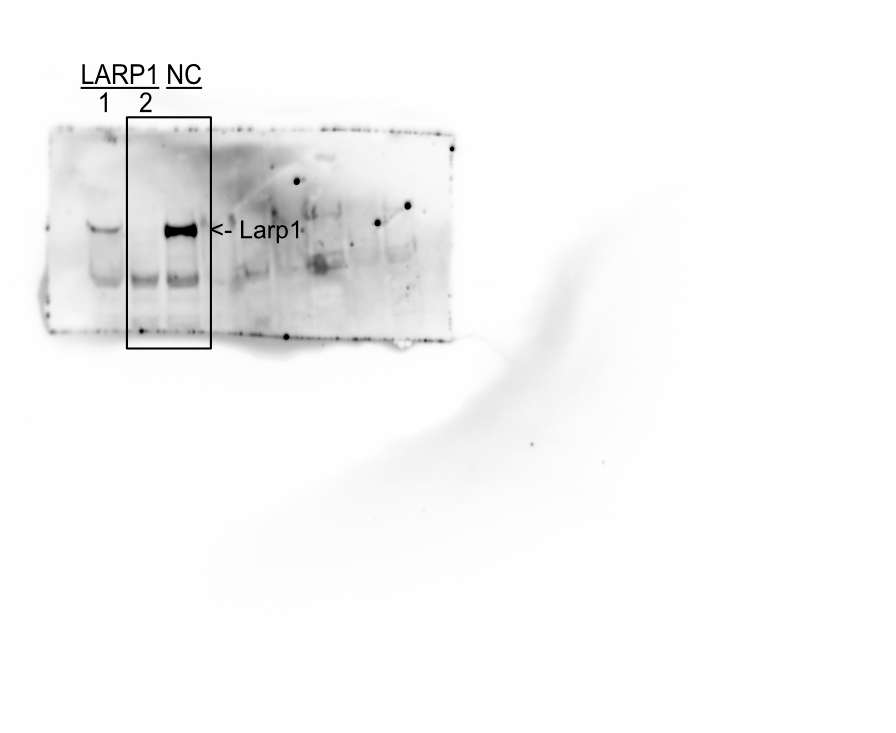

Supplement: Figure 4—source data 1. — LARP1 expression was visualized with a mouse monoclonal anti LARP1 antibody. [file elife-80040-fig4-data1.zip › figure4-sourcedata1/figure 4-source data 1-anno.tiff]

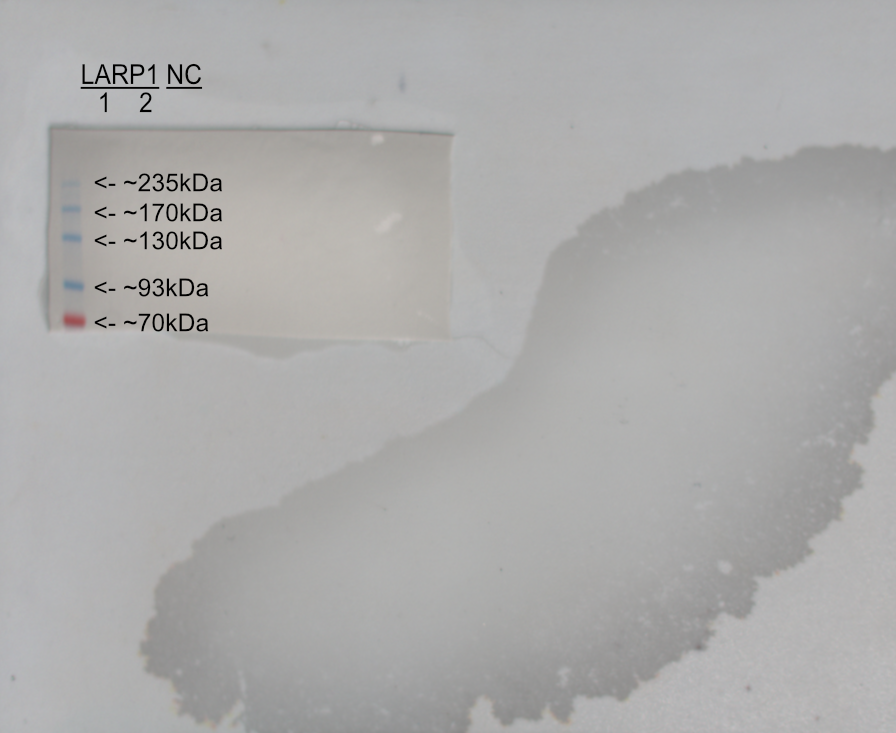

Supplement: Figure 4—source data 2. — The ladder is a broad-spectrum protein ladder (Fisher Scientific PI26623). [file elife-80040-fig4-data2.zip › figure4-sourcedata2/figure 4-source data 2-anno.tiff]

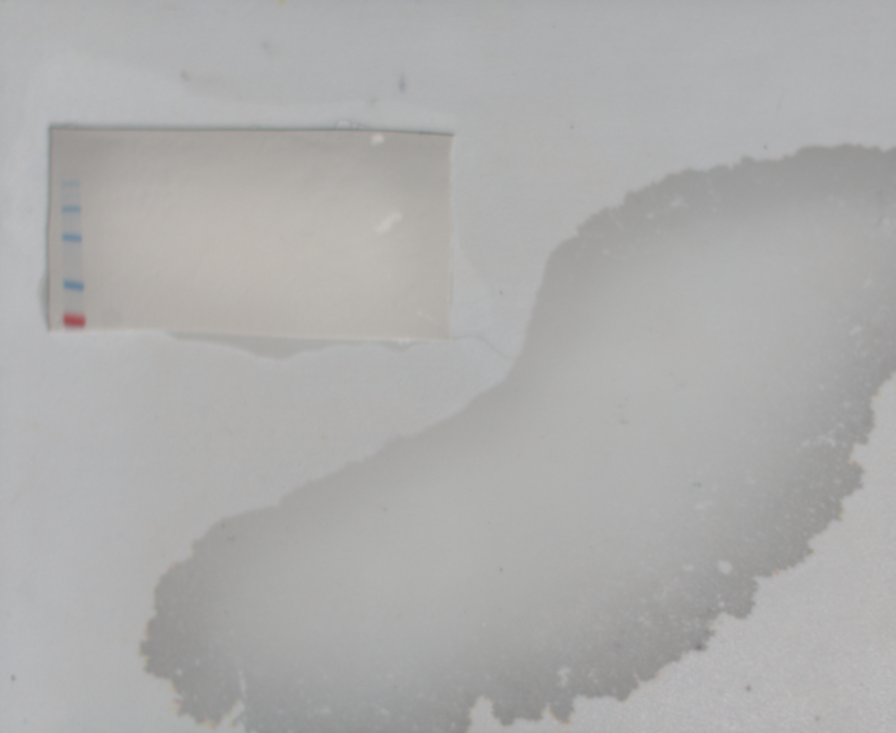

Supplement: Figure 4—source data 2. — The ladder is a broad-spectrum protein ladder (Fisher Scientific PI26623). [file elife-80040-fig4-data2.zip › figure4-sourcedata2/figure 4-source data 2.tif]

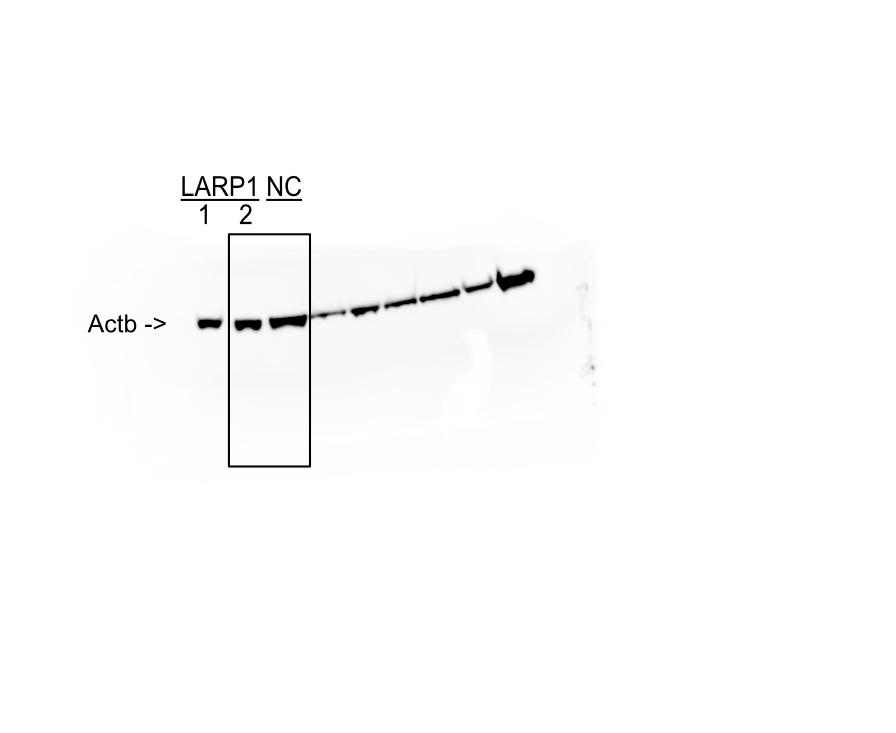

Supplement: Figure 4—source data 3. — Beta-actin expression was used as a loading control and visualized with a mouse monoclonal anti ACTB antibody. [file elife-80040-fig4-data3.zip › figure4-sourcedata3/figure 4-source data 3-anno.tiff]

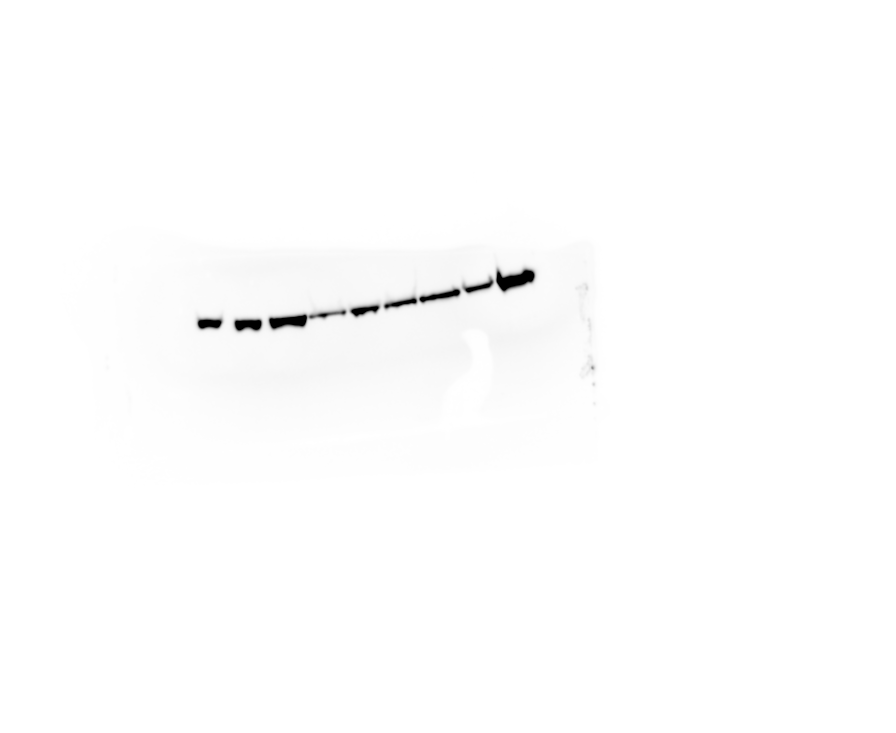

Supplement: Figure 4—source data 3. — Beta-actin expression was used as a loading control and visualized with a mouse monoclonal anti ACTB antibody. [file elife-80040-fig4-data3.zip › figure4-sourcedata3/figure 4-source data 3.tif]

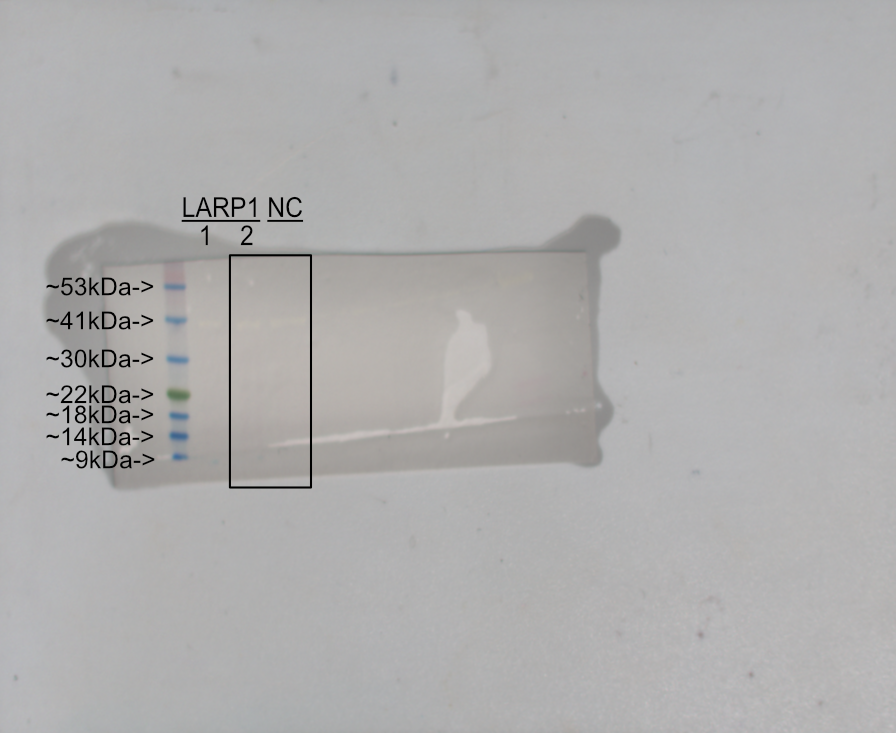

Supplement: Figure 4—source data 4. — The ladder is a broad-spectrum protein ladder (Fisher Scientific PI26623). [file elife-80040-fig4-data4.zip › figure4-sourcedata4/figure 4-source data 4-anno.tiff]

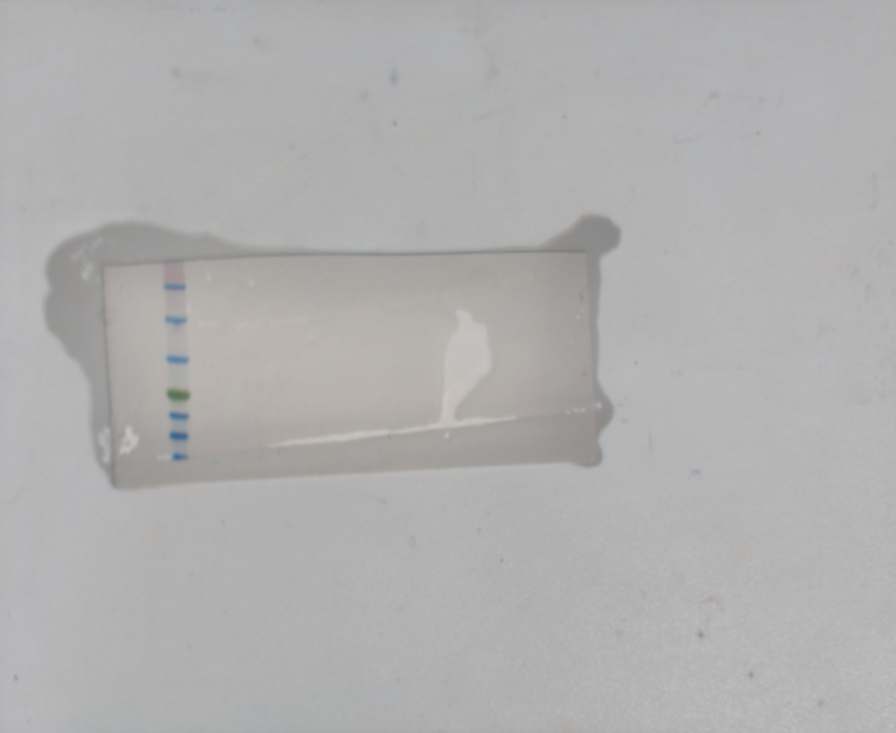

Supplement: Figure 4—source data 4. — The ladder is a broad-spectrum protein ladder (Fisher Scientific PI26623). [file elife-80040-fig4-data4.zip › figure4-sourcedata4/figure 4-source data 4.tif]

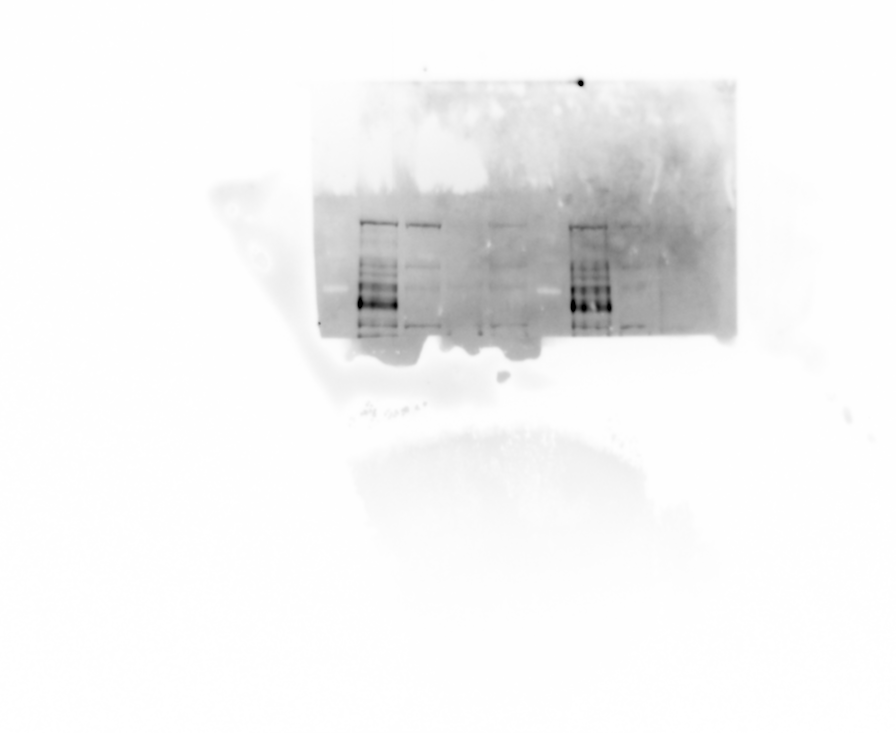

Supplement: Figure 4—source data 5. — LARP1 expression was visualized with a mouse monoclonal anti-LARP1 antibody. [file elife-80040-fig4-data5.zip › figure4-sourcedata5/figure 4-source data 5.tif]

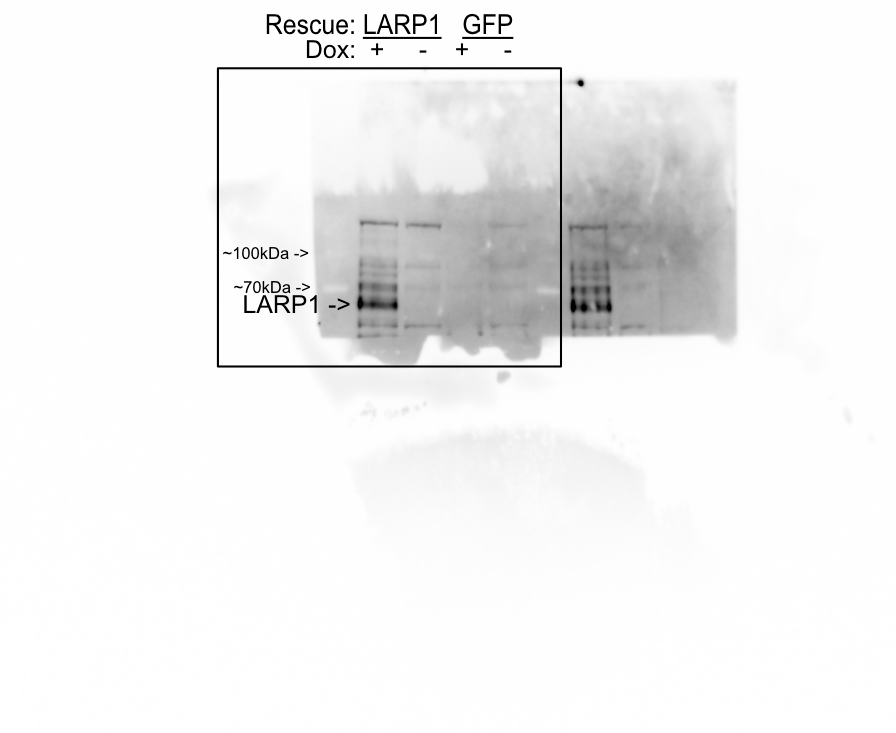

Supplement: Figure 4—source data 5. — LARP1 expression was visualized with a mouse monoclonal anti-LARP1 antibody. [file elife-80040-fig4-data5.zip › figure4-sourcedata5/figure 4-source data 5-anno.tiff]

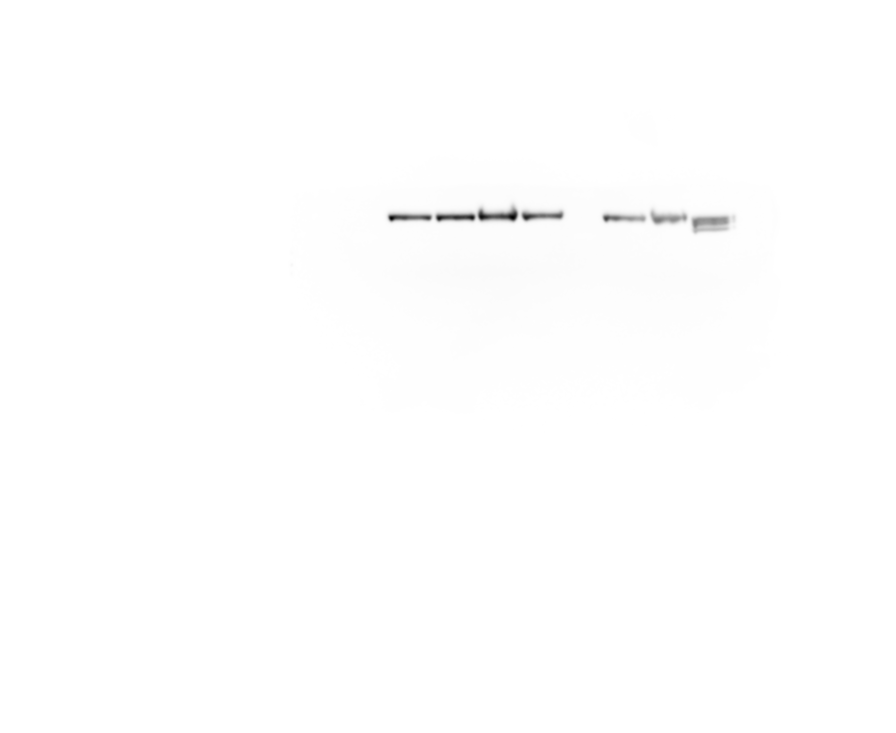

Supplement: Figure 4—source data 6. — Beta-actin expression was used as a loading control and visualized with a mouse monoclonal anti-ACTB antibody. [file elife-80040-fig4-data6.zip › figure4-sourcedata6/figure 4-source data 6.tif]

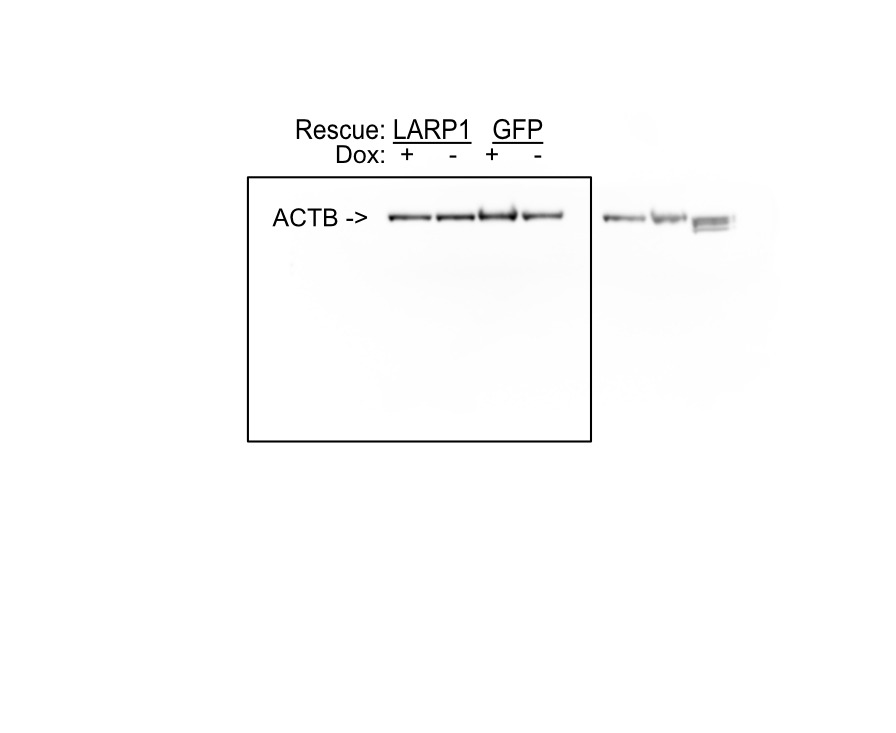

Supplement: Figure 4—source data 6. — Beta-actin expression was used as a loading control and visualized with a mouse monoclonal anti-ACTB antibody. [file elife-80040-fig4-data6.zip › figure4-sourcedata6/figure 4-source data 6-anno.tiff]

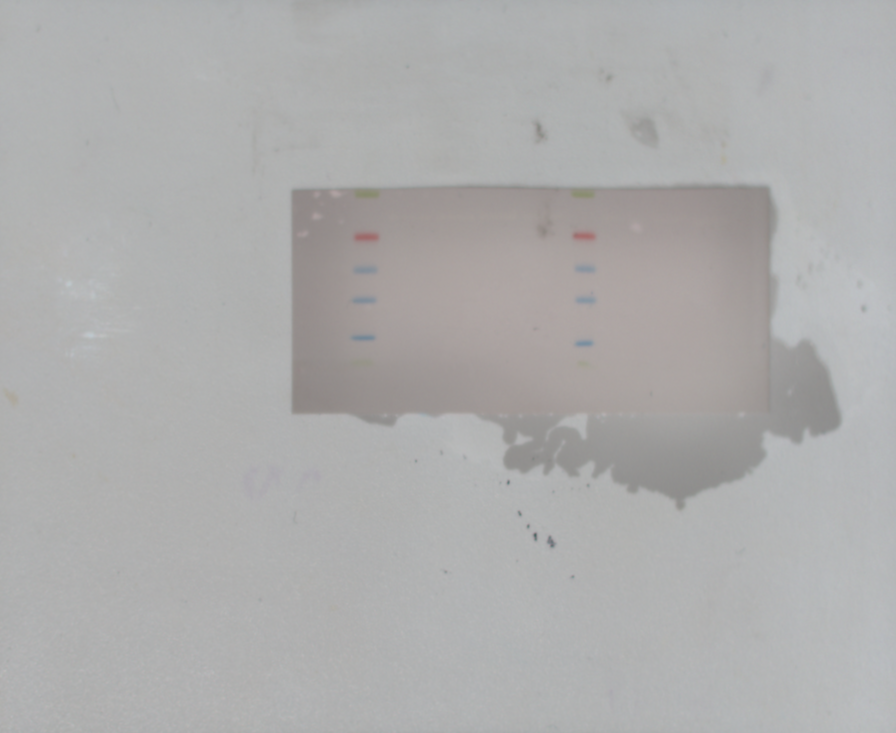

Supplement: Figure 4—source data 7. — The ladder is a broad-spectrum protein ladder (Fisher Scientific PI26623). [file elife-80040-fig4-data7.zip › figure4-sourcedata7/figure 4-source data 7.tif]

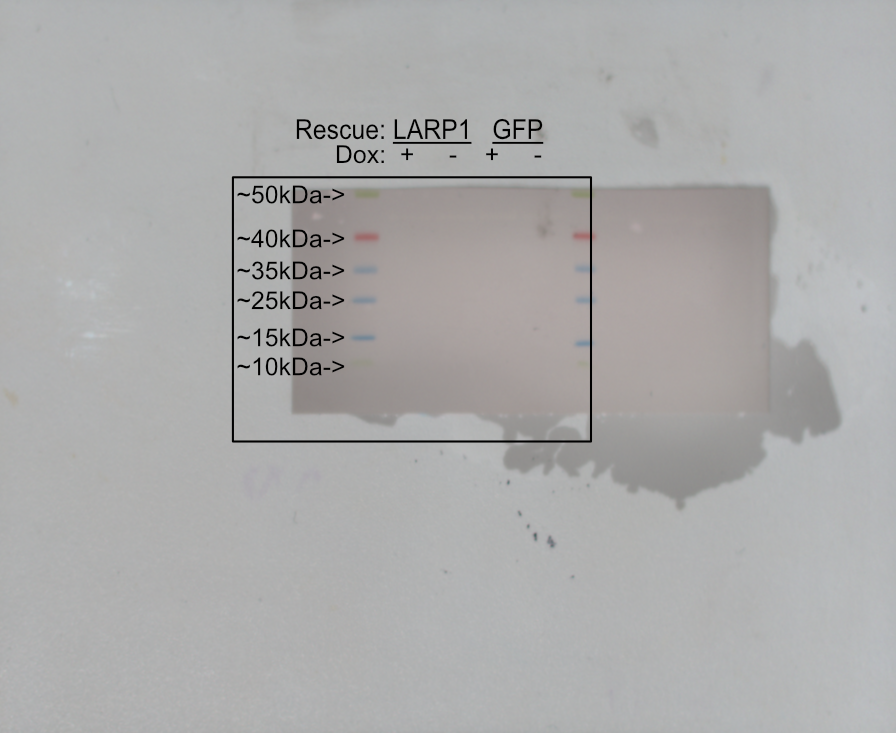

Supplement: Figure 4—source data 7. — The ladder is a broad-spectrum protein ladder (Fisher Scientific PI26623). [file elife-80040-fig4-data7.zip › figure4-sourcedata7/figure 4-source data 7-anno.tiff]

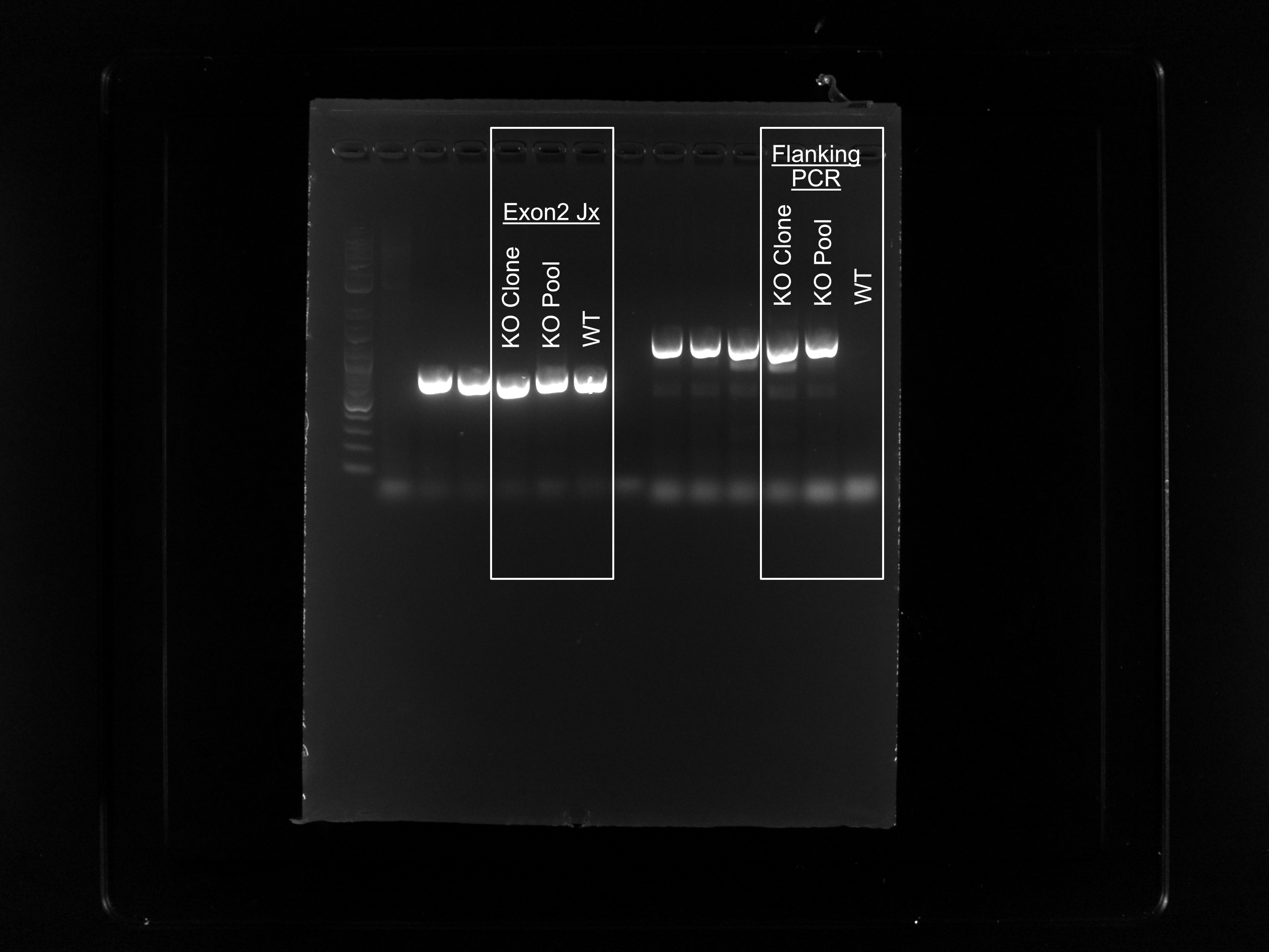

Supplement: Figure 4—figure supplement 7—source data 1. — DNA from several knockout clones, a heterogeneous pool of knockout cells and wildtype cells were assayed. The ladder used is the 1 kb Plus ladder (NEB N3200). [file elife-80040-fig4-figsupp7-data1.zip › figure4-figuresupp7-sourcedata1/figure 4-figure supplement 7-source data 1-anno.tiff]

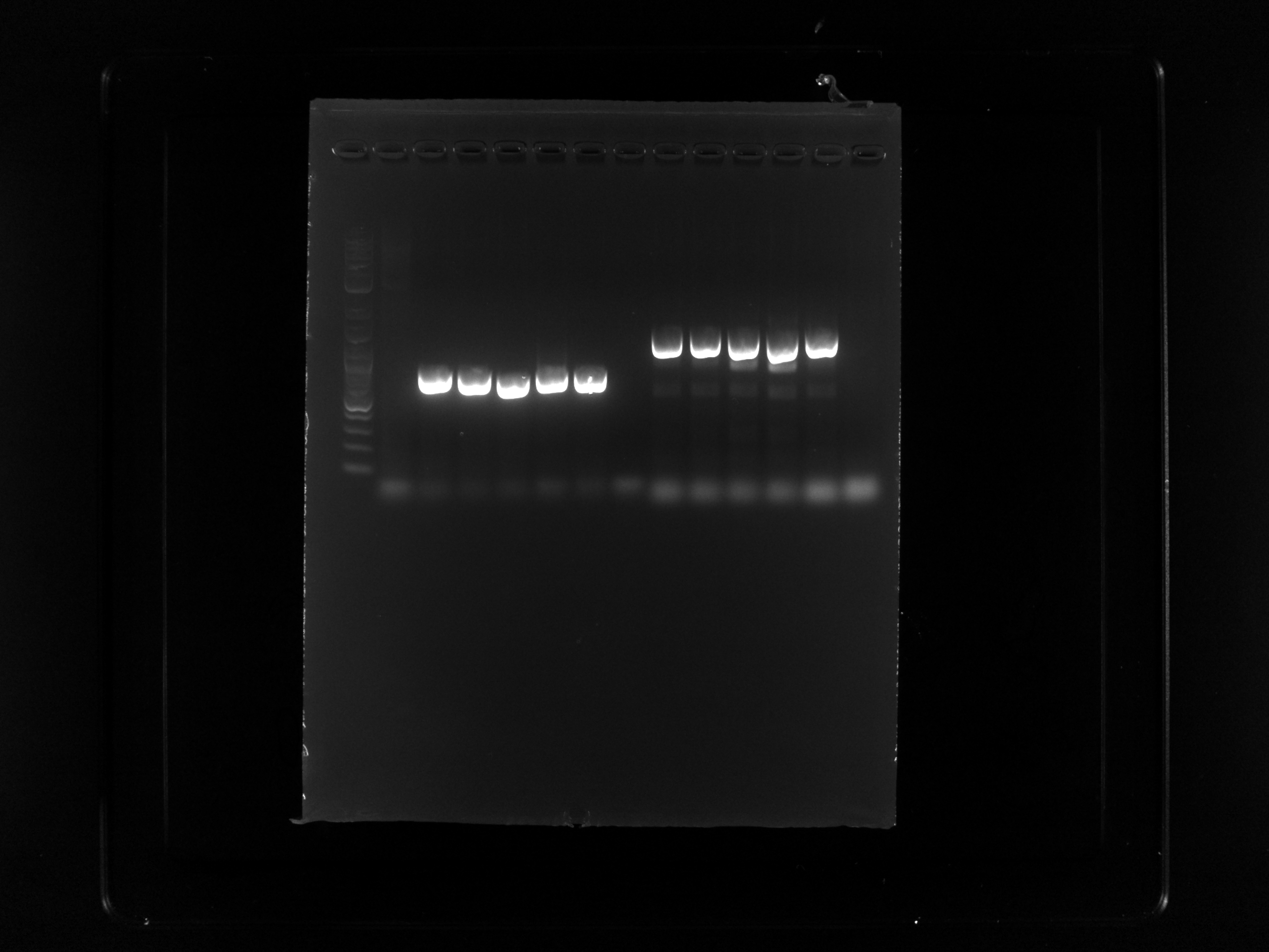

Supplement: Figure 4—figure supplement 7—source data 1. — DNA from several knockout clones, a heterogeneous pool of knockout cells and wildtype cells were assayed. The ladder used is the 1 kb Plus ladder (NEB N3200). [file elife-80040-fig4-figsupp7-data1.zip › figure4-figuresupp7-sourcedata1/figure 4-figure supplement 7-source data 1.tif]
